# Supplementary material for: Enhancing Gene Co-Expression Network Inference for the Malaria Parasite Plasmodium falciparum
Source: Genes (Basel). 2024 May 25;15(6):685. doi: 10.3390/genes15060685 (PMC11202799; doi:10.3390/genes15060685)
Supplement: Supplementary file 1 [file genes-15-00685-s001.zip › Malaria_MDPI_Gene_Supplement.pdf]

Supplementary material for: Enhancing gene co-expression network inference for the malaria parasite *Plasmodium falciparum*

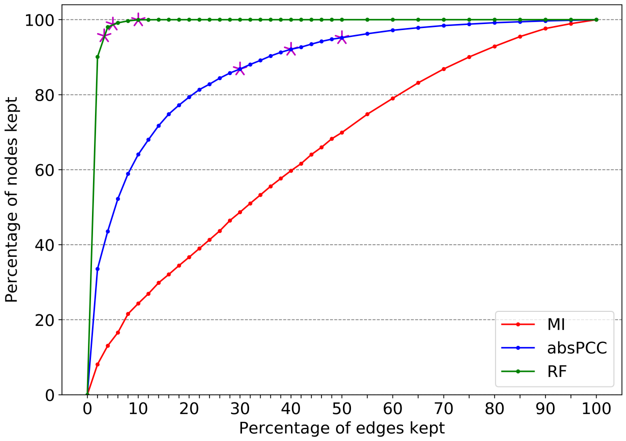

Supplementary Figure S1: The effect of edge weight thresholds on the size of the largest connected component for a given network. It illustrates the percent of nodes remained in the largest connected component out of all nodes in a given network when keeping $k\%$ of highest weighted edges in the given network (i.e., MI, absPCC, RF). The x-axis represents the percent of highest weighted edges kept (i.e., $k\%$) out of all edges in a given network. The y-axis represents the corresponding percent of nodes remained in the largest connected component out of all node in the gene expression data when keeping $k\%$ highest weighted edges. k is varied from 1 to 100. The magenta stars indicate the three thresholds we select for absPCC and RF, respectively. Note that we include MI in the figure as a control visualization for absPCC and RF.

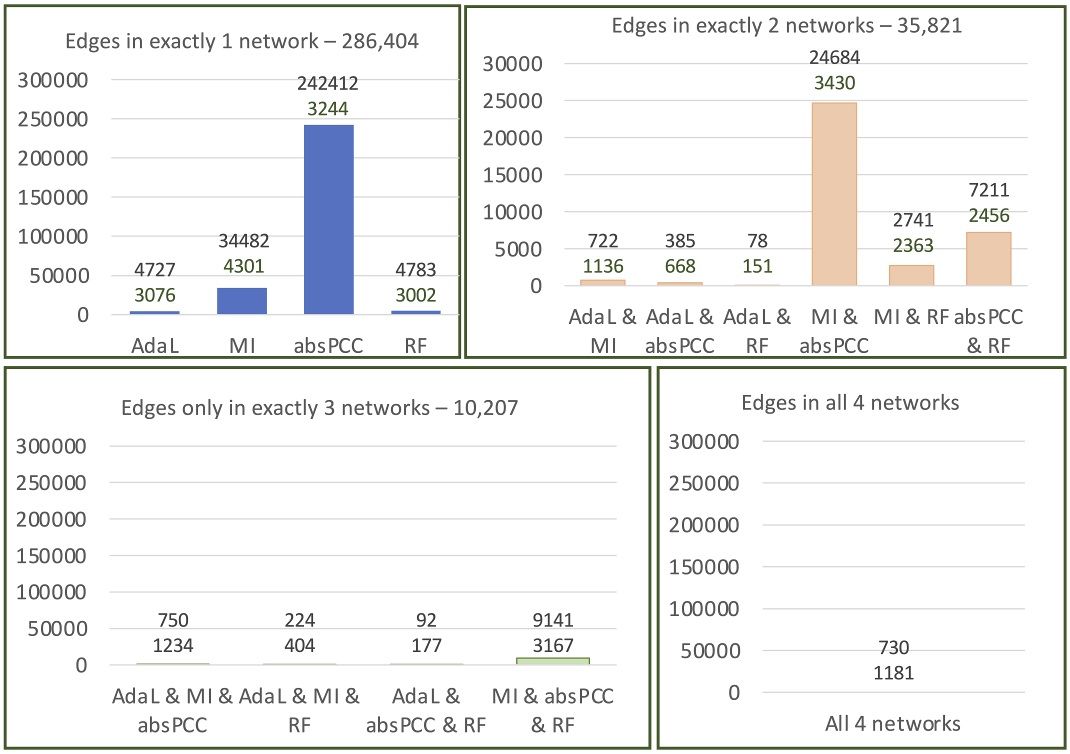

Supplementary Figure S2: Details about pairwise edge overlap between the four networks, i.e., MI, absPCC, RF, and AdaL. The box on the top left is the number of unique edges that are only present in exactly one network, where the x-axis shows the networks. The box on the top right is the number of unique edges that are only present in exactly two networks, where the x-axis shows the network pairs. The box on the left bottom is the number of unique edges that are only present in exactly three networks, where the x-axis shows the network triplets. The box on the right bottom is the number of unique edges that are present in all four networks, where the x-axis shows the four networks. All y-axes show the edge counts. The two numbers on top of each bar across the four boxes represent the number of edges and the number of nodes these edges encompass.

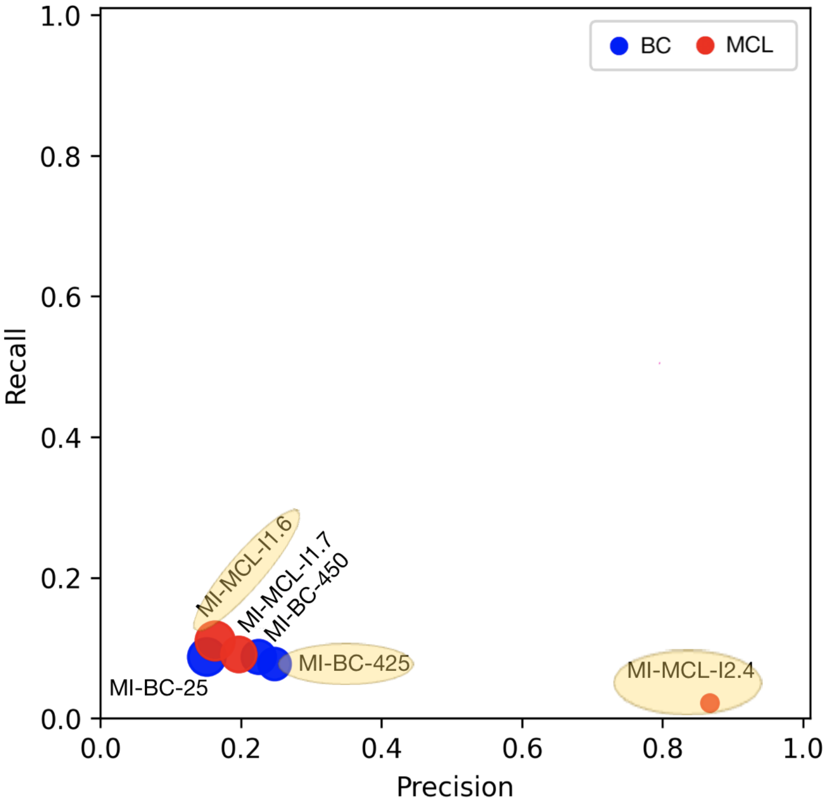

Supplementary Figure S3: Accuracy of predicting gene-GO term associations in the leave-one-out cross-validation in terms of precision and recall via MI. The sizes of the points correspond to the numbers of predictions produced by a given combination. The color of the points corresponds to a clustering method.

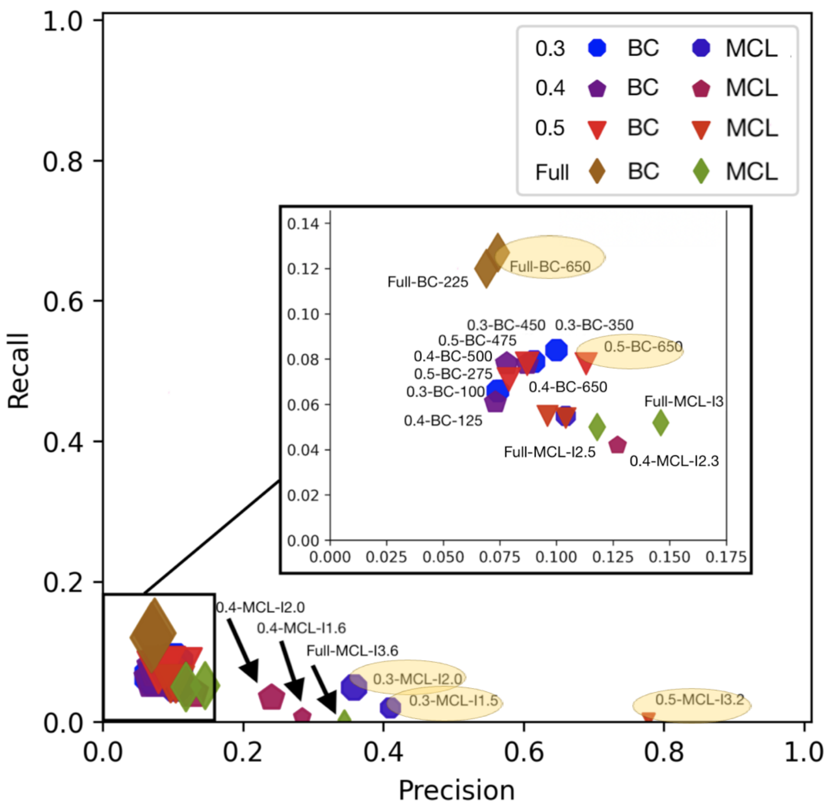

Supplementary Figure S4: Accuracy of predicting gene-GO term associations in the leave-one-out cross-validation in terms of precision and recall via absPCC. Each point is a combination of network, clustering method, and parameter value. The sizes of the points correspond to the number of predictions produced by a given combination. The shape of a point corresponds to a network, and the color shade of a point corresponds to a clustering method. For example, all circles correspond to absPCC-0.3, of which, light blue corresponds to BC and dark blue corresponds to MCL.

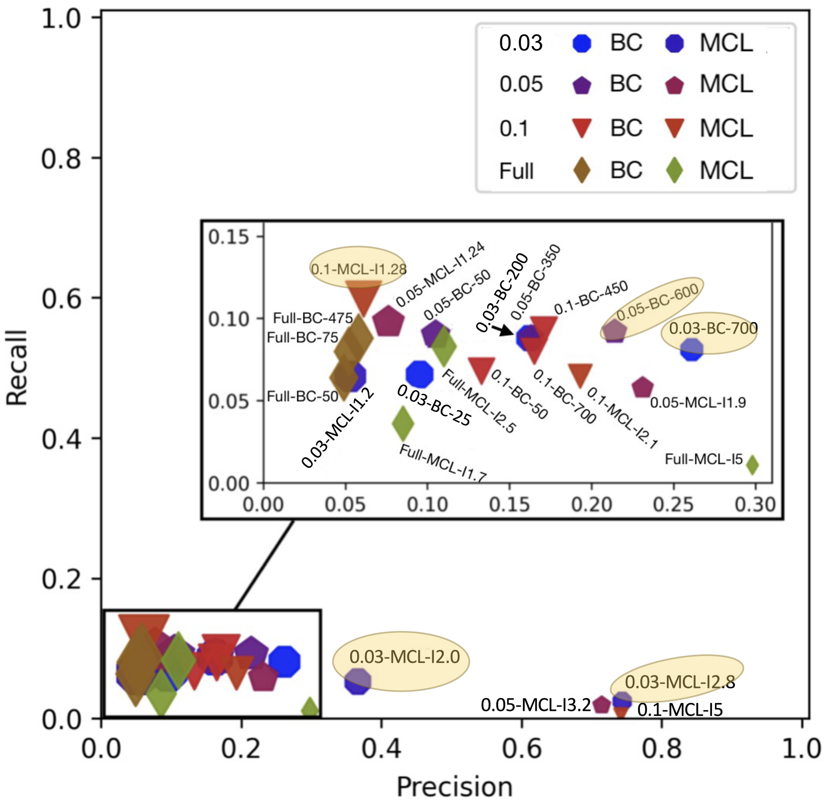

Supplementary Figure S5: Accuracy of predicting gene-GO term associations in the leave-one-out cross-validation in terms of precision and recall via RF. Each point is a combination of network, clustering method, and parameter value. The sizes of the points correspond to the number of predictions produced by a given combination. The shape of a point corresponds to a network, and the color shade of a point corresponds to a clustering method. For example, all circles correspond to RF-0.03, of which, light blue corresponds to BC and dark blue corresponds to MCL.

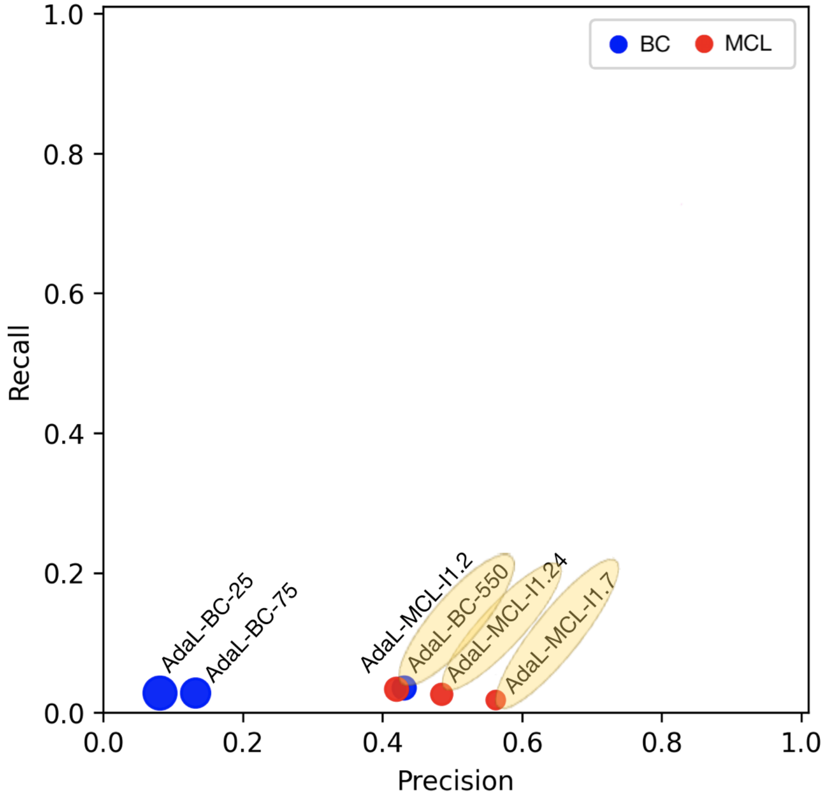

Supplementary Figure S6: Accuracy of predicting gene-GO term associations in the leave-one-out cross-validation in terms of precision and recall via AdaL. The sizes of the points correspond to the number of predictions produced by a given combination. The color of the points corresponds to a clustering method.

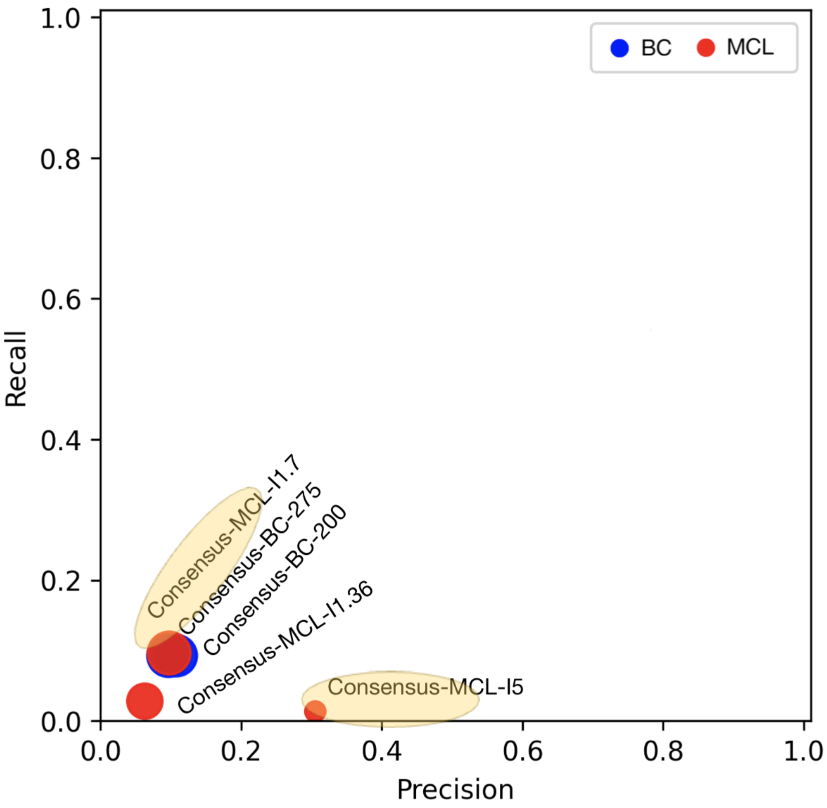

Supplementary Figure S7: Accuracy of predicting gene-GO term associations in the leave-one-out cross-validation in terms of precision and recall via Consensus. The sizes of the points correspond to the number of predictions produced by a given combination. The color of the points corresponds to a clustering method.

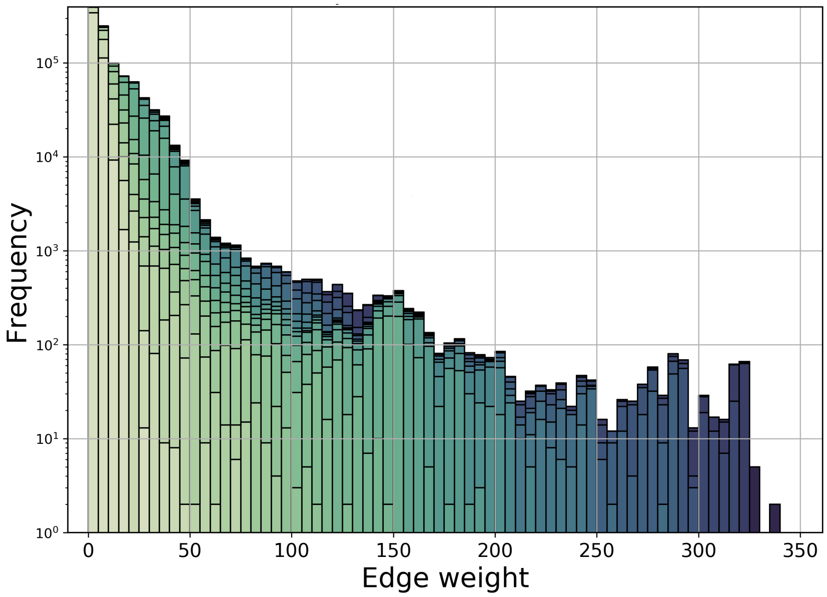

Supplementary Figure S8: The distribution of confidence scores for predicted gene-gene interactions. The color shades represent the number of combinations of a network and clustering method that support the corresponding association. The darker color the color, the higher the support. Analogous results for gene-GO term associations are shown in Figure 7 of the main paper.
